# Molecular and Treatment-Associated Correlates of Survival in Glioblastoma: Complementary Surveillance, Epidemiology, and End Results (SEER) and The Cancer Genome Atlas-Glioblastoma (TCGA-GBM) Cohort Analyses

**DOI:** 10.7759/cureus.112222

**Published:** 2026-07-07

**Authors:** Kevin Woodson

**Affiliations:** 1 Internal Medicine, Independent Research, Norman, USA

**Keywords:** glioblastoma, idh-wildtype glioma, mgmt methylation, seer program, the cancer genome atlas

## Abstract

Background

Glioblastoma (GBM) carries a poor prognosis. We evaluated population-level and tumor-level determinants of survival in parallel Surveillance, Epidemiology, and End Results (SEER) and The Cancer Genome Atlas-Glioblastoma (TCGA-GBM) cohorts.

Methods

SEER cases (histology 9440/3, 2000-2022) were stratified by diagnosis era and registry-recorded trimodality therapy. TCGA-GBM IDH-wildtype cases were analyzed by MGMT promoter methylation status. Kaplan-Meier, log-rank, and multivariable Cox models were applied.

Results

SEER included 53,432 cases (48,715 deaths), and TCGA IDH-wildtype included 512 cases. Median overall survival (OS) rose modestly across eras (7.0, 9.0, 10.0 months) and remained independently associated with survival after adjustment. Recorded trimodality therapy was associated with longer median OS (14.0 vs 4.0 months). In TCGA, median OS was 15.7 vs 12.5 months for MGMT-methylated vs unmethylated GBM (p=0.012), but MGMT methylation was not significant after multivariable adjustment (HR 0.83, 95% CI 0.63-1.08, p=0.162).

Conclusions

Era-associated survival gains persisted after adjustment, suggesting that multimodality therapy uptake explains only part of population-level improvements. MGMT methylation predicted unadjusted but not adjusted survival in IDH-wildtype GBM, consistent with known age confounding.

## Introduction

Glioblastoma (GBM) carries a poor prognosis and is the most common malignant primary brain tumor in adults. Despite maximal safe resection followed by concurrent and adjuvant temozolomide with radiotherapy (the Stupp protocol) [1], median overall survival (OS) remains 12-18 months, and five-year survival is below 10%. Three molecular features have reshaped modern GBM care. First, MGMT promoter methylation silences the DNA repair enzyme O6-methylguanine DNA methyltransferase and is associated with benefit from temozolomide [2]. Second, IDH1/IDH2 mutations now define a separate astrocytoma lineage under the 2021 WHO classification [3]. By exclusion, this refines the diagnosis of GBM to IDH-wildtype disease. Third, transcriptional subtypes (classical, mesenchymal, proneural) described by Verhaak and colleagues [4] have less consistent treatment implications. Rather than treating registry trends and tumor biomarkers as separate questions, this analysis juxtaposes population-level treatment-era outcomes with tumor-level molecular stratification to contextualize the relative magnitude of clinical treatment proxies and MGMT-associated survival heterogeneity in IDH-wildtype GBM. Specifically, we used Surveillance, Epidemiology, and End Results (SEER) to evaluate population-level, treatment-era, and demographic correlates of OS, and The Cancer Genome Atlas-Glioblastoma (TCGA-GBM) to evaluate tumor-level molecular correlates (MGMT promoter methylation and Verhaak transcriptional subtype) within the WHO 2021 IDH-wildtype category. We hypothesized that population-level survival gains across diagnosis eras would be only partially explained by recorded multimodality therapy receipt and that the MGMT-survival association in unadjusted IDH-wildtype TCGA-GBM analyses would attenuate after adjustment for age and subtype.

## Materials and methods

SEER cohort

Cases with ICD-O-3 histology 9440/3 (glioblastoma, NOS) diagnosed 2000-2022 were extracted from the SEER Research Database [5]. Cases with missing or zero recorded survival time were excluded. Diagnosis era was coded as pre-Stupp (2000-2004), Stupp era (2005-2014) [1], and TTFields era (2015-2022) [6]. These labels reflect the calendar period and not the confirmed individual receipt of either temozolomide or TTFields. Surgery was defined as any recorded resection or excision in the SEER surgery code (codes 10-90; code 00 = no surgery). Radiation was defined as any recorded radiation other than 'None,' 'Refused,' or 'Unknown.' Chemotherapy was defined as a 'Yes' on the SEER chemotherapy recode. A registry-recorded trimodality-therapy flag was set when surgery, radiation, and chemotherapy were all recorded as received; this is a SEER-based proxy and does not confirm Stupp-protocol receipt, dose, or completion. Overall survival was measured in months from the date of diagnosis to the date of death (event) or the date of last known contact (censored), as recorded in the SEER 'Survival months' variable; events were vital-status deaths. Patients with unknown race were excluded from the multivariable Cox model because race was included as a covariate. A multivariable Cox model was fit with age group (collapsed from SEER 5-year bins to 18-44, 45-64, 65-74, 75+), sex, race, surgery, radiation, chemotherapy, and era as covariates; reference categories were age <45 years, female sex, White race, no recorded surgery, no recorded radiation, no recorded chemotherapy, and diagnosis era 2000-2004.

TCGA cohort

The gbm_tcga_pub2013 study [7] was obtained from cBioPortal [8]. We selected this study rather than the more recent gbm_tcga_pan_can_atlas_2018 release because MGMT promoter methylation status, the primary tumor-level exposure of this analysis, is exposed as a clinical attribute in the Cell 2013 study but is not available as a clinical attribute in the PanCancer Atlas 2018 release. For the primary analysis, we applied the WHO 2021 definition [3] by retaining IDH-wildtype cases only. The primary exposure was MGMT promoter methylation status (methylated versus unmethylated) [2]; cases with missing MGMT status were excluded from the primary KM and Cox analyses. A sensitivity KM analysis on the historical TCGA-GBM cohort, including IDH-mutant cases, is reported separately. A multivariable Cox model adjusted for age (continuous), sex, and Verhaak transcriptional subtype [4] where available (Classical, Mesenchymal, Proneural, or Neural); reference categories were MGMT-unmethylated, female sex, and Classical subtype. A small number of G-CIMP cases were excluded from the multivariable model because G-CIMP is a methylation-defined phenotype rather than a Verhaak transcriptional class and is strongly associated with IDH-mutant tumors (already excluded by the WHO 2021 filter). Of the 512 IDH-wildtype patients, 323 had MGMT promoter methylation status available for the primary Kaplan-Meier (KM) analysis; 319 had complete covariates (MGMT, age, sex, Verhaak subtype) for the multivariable Cox model. SEER data were accessed via SEER*Stat on May 3, 2026; TCGA-GBM data were accessed via cBioPortal on May 3, 2026. Both datasets are de-identified by the data providers (SEER Program and TCGA Network, respectively); the author had no access to direct or indirect personally identifying information at any point during data acquisition or analysis.

Statistics

Analyses were performed in Python using lifelines and matplotlib. KM estimates were compared between groups by the log-rank test and displayed truncated at 60 months for visualization. KM, log-rank, and Cox proportional hazards analyses were implemented with standard survival-analysis methods, with a small ridge penalty applied to the Cox models. Two-sided p-values < 0.05 were considered significant. No adjustment for multiplicity was performed given the pre-specified two-cohort design. Proportional hazards assumptions were not formally tested; results should be interpreted as average hazard ratios over follow-up.

## Results

SEER overall survival by era and recorded treatment

**Figure 1:**
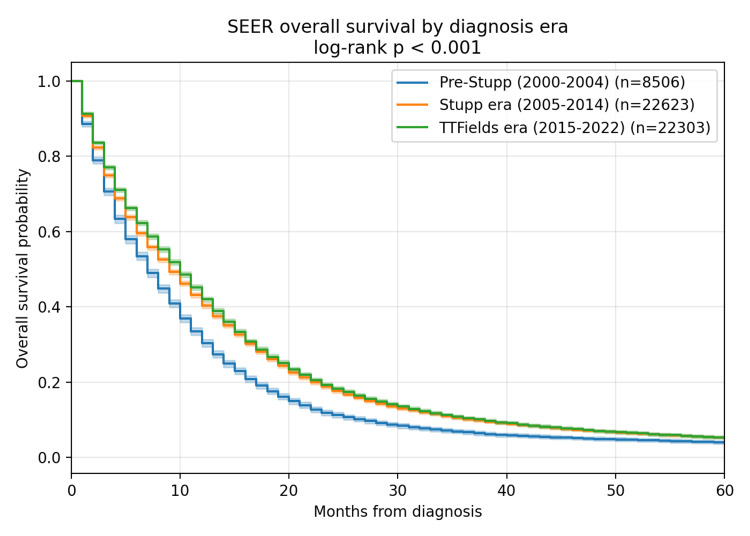
SEER overall survival by diagnosis era. Median OS: 7.0 months (pre-Stupp), 9.0 months (2005-2014), 10.0 months (2015-2022); log-rank p < 0.001. SEER: Surveillance, Epidemiology, and End Results; OS: Overall Survival

**Figure 2:**
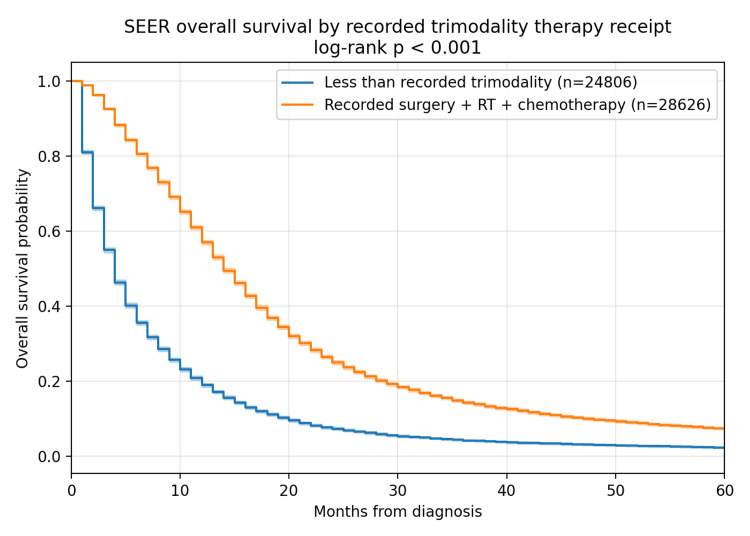
SEER overall survival by registry-recorded trimodality therapy (surgery + radiation + chemotherapy). Median OS: 14.0 versus 4.0 months; log-rank p < 0.001. SEER: Surveillance, Epidemiology, and End Results; OS: Overall Survival

**Figure 3:**
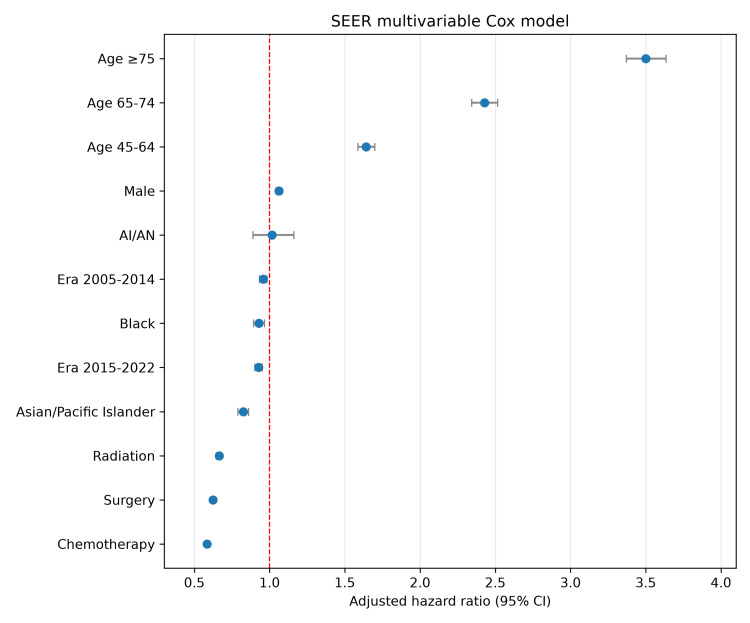
SEER multivariable Cox hazard ratios (95% CI). Forest plot of adjusted hazard ratios from the SEER multivariable Cox model (n = 53,432). Points represent hazard ratios and horizontal bars represent 95% confidence intervals. Reference: age <45 years, female sex, White race, no recorded surgery, no recorded radiation, no recorded chemotherapy, and diagnosis era 2000-2004. SEER: Surveillance, Epidemiology, and End Results

Demographic and clinical characteristics of the SEER and TCGA-GBM cohorts are summarized in Table 1. A total of 58,506 cases met initial inclusion criteria (ICD-O-3 9440/3, age ≥18, diagnosis 2000-2022). After excluding 4,929 cases with missing or zero recorded survival months and 145 with unknown race, the final SEER cohort comprised 53,432 patients with 48,715 deaths. Median OS by diagnosis era rose modestly from 7.0 months (pre-Stupp) to 9.0 months (2005-2014) and 10.0 months (2015-2022), with log-rank p < 0.001 (Figure 1). Patients with registry-recorded trimodality therapy (surgery + radiation + chemotherapy) had longer median OS (14.0 months) than those with less than recorded trimodality (4.0 months; p < 0.001; Figure 2). The multivariable Cox model included n = 53,432 patients after exclusion of cases with unknown race (Figure 3; Table 2); advancing age and absence of any recorded modality of therapy (surgery, radiation, or chemotherapy) carried the largest hazards. The independent association of each individual treatment modality with overall survival is estimated in the multivariable Cox model (Table 2): adjusted HRs for chemotherapy, surgery, and radiation receipt versus none were 0.58 (95% CI 0.57-0.60), 0.62 (95% CI 0.61-0.64), and 0.66 (95% CI 0.65-0.68), respectively, all p < 0.001. Importantly, the era effect was attenuated but persisted after adjustment for age, sex, race, and recorded treatment receipt: 2005-2014 vs 2000-2004 carried HR 0.96 (95% CI 0.93-0.98), p = 0.001, and 2015-2022 vs 2000-2004 carried HR 0.93 (95% CI 0.90-0.95), p < 0.001.

**Table 1:** Demographic and clinical characteristics of the SEER and TCGA-GBM cohorts. SEER cohort reflects ICD-O-3 histology 9440/3 cases diagnosed 2000-2022 after exclusion of cases with missing/zero survival time and unknown race. TCGA-GBM IDH-wildtype cohort reflects application of the WHO 2021 definition; 323 of these 512 patients had MGMT promoter methylation status available for the primary Kaplan-Meier analysis, and 319 had complete covariates (MGMT, age, sex, Verhaak subtype) for the multivariable Cox model. Race is not analyzed in TCGA because of limited and heterogeneous race recording across contributing centers. G-CIMP cases are excluded from the multivariable Cox model. SEER: Surveillance, Epidemiology, and End Results; TCGA-GBM: The Cancer Genome Atlas-Glioblastoma

Characteristic	SEER cohort (n=53,432)	TCGA-GBM IDH-wildtype cohort (n=512)
Age		Median 60.2 years (IQR 51.1-69.2; range 10.9-89.3)
18-44 years	4,155 (7.8%)	66 (12.9%)
45-64 years	22,828 (42.7%)	264 (51.6%)
65-74 years	15,162 (28.4%)	114 (22.3%)
≥75 years	11,287 (21.1%)	68 (13.3%)
Sex		
Female	22,388 (41.9%)	199 (38.9%)
Male	31,044 (58.1%)	313 (61.1%)
Race		Not analyzed
White	47,533 (89.0%)	—
Black	3,013 (5.6%)	—
Asian or Pacific Islander	2,645 (5.0%)	—
American Indian/Alaska Native	241 (0.5%)	—
Diagnosis era		Not applicable
2000-2004	8,506 (15.9%)	—
2005-2014	22,623 (42.3%)	—
2015-2022	22,303 (41.7%)	—
Registry-recorded treatment		Not applicable
Surgery	41,627 (77.9%)	—
Radiation	40,309 (75.4%)	—
Chemotherapy	34,820 (65.2%)	—
Trimodality (surgery + RT + chemo)	28,626 (53.6%)	—
MGMT promoter methylation	Not captured	
Methylated	—	148 (28.9%)
Unmethylated	—	175 (34.2%)
Missing/Unknown	—	189 (36.9%)
Verhaak transcriptional subtype	Not captured	
Classical	—	146 (28.5%)
Mesenchymal	—	156 (30.5%)
Proneural	—	98 (19.1%)
Neural	—	82 (16.0%)
G-CIMP	—	12 (2.3%)
Missing	—	18 (3.5%)
Vital status		
Deceased	48,715 (91.2%)	400 (78.1%)
Alive (censored)	4,717 (8.8%)	112 (21.9%)
Median overall survival	9.0 months	10.6 months

**Table 2:** SEER multivariable Cox model. Adjusted hazard ratios from the SEER multivariable Cox model. All variables shown are mutually adjusted. SEER: Surveillance, Epidemiology, and End Results

Variable (vs reference)	HR	95% CI	p-value
Chemotherapy recorded vs none	0.58	0.57-0.60	< 0.001
Surgery recorded vs none	0.62	0.61-0.64	< 0.001
Radiation recorded vs none	0.66	0.65-0.68	< 0.001
Asian/Pacific Islander vs White	0.82	0.79-0.86	< 0.001
2015-2022 vs 2000-2004	0.93	0.90-0.95	< 0.001
Black vs White	0.93	0.89-0.96	< 0.001
2005-2014 vs 2000-2004	0.96	0.93-0.98	0.001
AI/AN vs White	1.02	0.89-1.16	0.813
Male vs female	1.06	1.04-1.08	< 0.001
Age 45-64 vs <45	1.64	1.59-1.70	< 0.001
Age 65-74 vs <45	2.43	2.34-2.52	< 0.001
Age >=75 vs <45	3.50	3.37-3.63	< 0.001

TCGA molecular features and survival

**Figure 4:**
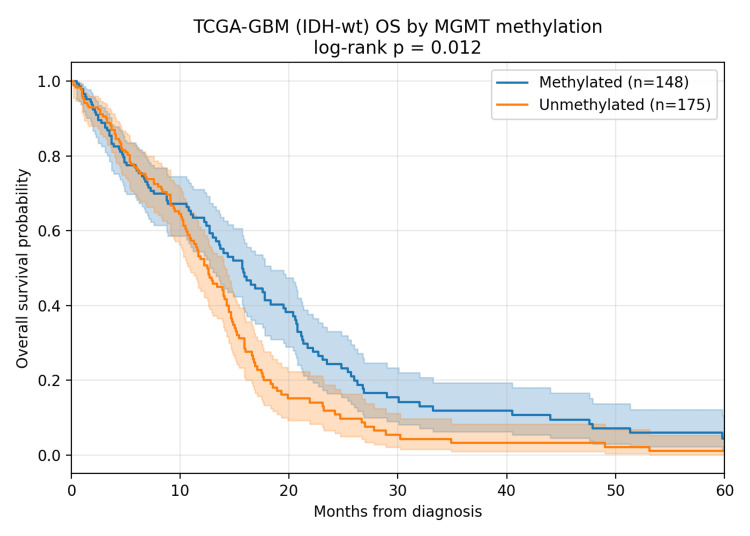
TCGA-GBM (IDH-wildtype) Kaplan-Meier curves by MGMT promoter methylation. Median OS: 15.7 months (methylated) versus 12.5 months (unmethylated); log-rank p = 0.012. TCGA-GBM: The Cancer Genome Atlas-Glioblastoma; OS: Overall Survival

**Figure 5:**
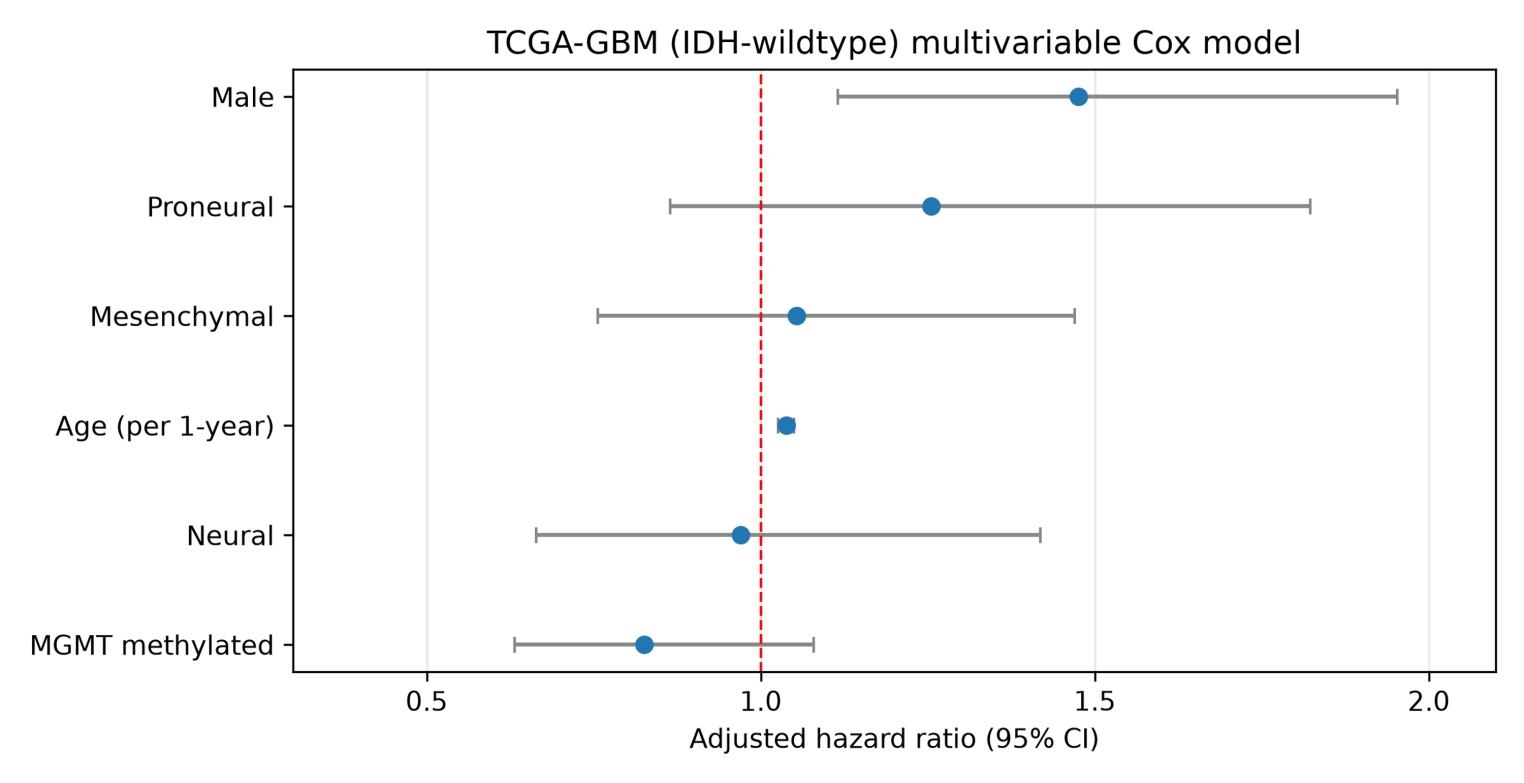
TCGA-GBM (IDH-wildtype) multivariable Cox hazard ratios (95% CI). Forest plot of adjusted hazard ratios from the TCGA-GBM (IDH-wildtype) multivariable Cox model (n = 319). Points represent hazard ratios and horizontal bars represent 95% confidence intervals. Reference: MGMT unmethylated, female sex, Classical Verhaak subtype TCGA-GBM: The Cancer Genome Atlas-Glioblastoma

After applying the WHO 2021 filter [3], the TCGA-GBM cohort contained 512 IDH-wildtype patients. Of these, 323 had MGMT promoter methylation status available for the primary KM analysis and 319 had complete covariates (MGMT, age, sex, subtype) for the multivariable Cox model. MGMT-methylated tumors had median OS of 15.7 months versus 12.5 months for MGMT-unmethylated tumors (log-rank p = 0.012; Figure 4) [2]. In the multivariable Cox model (n = 319 after exclusion of cases with missing covariates) adjusting for age, sex, and Verhaak subtype [4], the association was attenuated and no longer statistically significant after adjustment (adjusted HR 0.83 (95% CI 0.63-1.08), p = 0.162; Figure 5; Table 3).

**Table 3:** TCGA multivariable Cox model. Adjusted hazard ratios from the TCGA-GBM (IDH-wildtype) multivariable Cox model. All variables shown are mutually adjusted. TCGA-GBM: The Cancer Genome Atlas-Glioblastoma

Variable (vs reference)	HR	95% CI	p-value
MGMT methylated vs unmethylated	0.83	0.63-1.08	0.162
Neural vs Classical	0.97	0.66-1.42	0.876
Age (per one-year increase)	1.04	1.03-1.05	< 0.001
Mesenchymal vs Classical	1.05	0.76-1.47	0.757
Proneural vs Classical	1.25	0.86-1.82	0.233
Male vs female	1.48	1.11-1.95	0.006

Sensitivity analysis: TCGA Cox model with TMZ-containing therapy as a covariate

Because the TCGA-GBM clinical therapy field is heterogeneous (15 distinct categories, including 18 patients labeled "Unspecified Therapy"), it was not included in the primary Cox model. In a sensitivity analysis, we added a binary covariate for TMZ-containing therapy (any recorded TMZ use vs no recorded TMZ) to the IDH-wildtype multivariable Cox model after excluding patients with Unspecified Therapy, yielding a model of n = 307. TMZ-containing therapy was independently associated with a substantially lower hazard (adjusted HR 0.45, 95% CI 0.34-0.61, p < 0.001). With TMZ included, the adjusted MGMT effect was further attenuated (HR 0.90, 95% CI 0.68-1.19, p = 0.457), consistent with part of the MGMT-associated survival signal being mediated through TMZ receipt and benefit. The direction and magnitude of the age, sex, and Verhaak subtype effects were materially unchanged from the primary model (Table 4). These estimates should be interpreted cautiously: receipt of TMZ-containing therapy in TCGA is itself confounded with age, performance status, and other unmeasured factors that determine treatment selection.

**Table 4:** TCGA-GBM (IDH-wildtype) multivariable Cox model — sensitivity analysis including TMZ-containing therapy as a covariate. Adjusted hazard ratios from the multivariable Cox model with TMZ-containing therapy added as a binary covariate. Reference categories: MGMT-unmethylated, no/non-TMZ therapy, female sex, Classical Verhaak subtype. Patients with Unspecified Therapy (n=18) and any with missing covariates were excluded; n=307 patients entered the model. TCGA-GBM: The Cancer Genome Atlas-Glioblastoma

Variable (vs reference)	HR	95% CI	p-value
TMZ-containing therapy vs no/non-TMZ	0.45	0.34-0.61	< 0.001
MGMT methylated vs unmethylated	0.9	0.68-1.19	0.457
Age (per one-year increase)	1.03	1.02-1.04	< 0.001
Mesenchymal vs Classical	1.09	0.78-1.53	0.617
Neural vs Classical	1.09	0.73-1.62	0.675
Proneural vs Classical	1.21	0.83-1.78	0.318
Male vs female	1.69	1.27-2.26	< 0.001

Sensitivity analysis: historical TCGA cohort by IDH status

**Figure 6:**
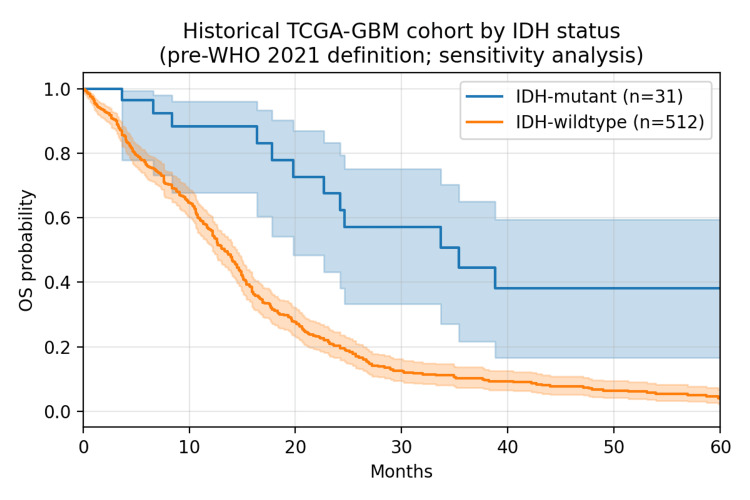
Historical TCGA-GBM cohort overall survival by IDH status. Sensitivity analysis only; IDH-mutant tumors are no longer classified as glioblastoma under WHO 2021 and were excluded from the primary cohort. TCGA-GBM: The Cancer Genome Atlas-Glioblastoma; OS: Overall Survival

In the historical TCGA-GBM cohort (pre-WHO 2021 definition, including IDH-mutant cases), KM curves stratified by IDH status reproduce the well-known IDH-mutant survival advantage; IDH-mutant tumors are now classified as astrocytoma rather than glioblastoma under WHO 2021 [3] and were excluded from the primary analysis (Figure 6).

## Discussion

These parallel cohort analyses highlight two complementary patterns. First, in SEER, median OS rose modestly across successive diagnosis eras coinciding with publication of the Stupp protocol and approval of TTFields. After adjustment for age, sex, race, and recorded treatment receipt, the era effect was attenuated but remained statistically significant, indicating that uptake of recorded multimodality therapy explains part but not all of the population-level gains. The magnitude of population-level era gains in our SEER cohort is consistent with, but smaller than, the survival benefits seen in the long-term follow-up of the pivotal temozolomide trial [9]. Neither SEER nor TCGA captures performance status, temozolomide dosing or cycle completion, or radiotherapy fractions, so the contribution of dosing intensity to this gap cannot be assessed directly from these datasets. The divergence between registry-based and trial-based outcomes likely reflects several mutually reinforcing factors: incomplete real-world uptake of full Stupp-protocol dosing, the inclusion in unselected population-based cohorts of older, frailer, and lower-performance-status patients who would have been ineligible for the pivotal trials, less rigorous follow-up, and later detection of treatment-related complications. Plausible additional contributors include improvements in surgical technique, such as fluorescence-guided resection [10] and the well-described association of greater extent of resection with longer survival [11], supportive care, neuro-oncology subspecialty access, and post-progression therapy that are not captured by the SEER treatment fields [12]. Importantly, registry-recorded trimodality therapy in SEER is itself a proxy: it does not confirm concurrent and adjuvant temozolomide, RT dose, surgical extent, TTFields use, or performance status, all of which are likely confounded with treatment receipt.

Second, in TCGA, MGMT promoter methylation stratifies tumors with different unadjusted survival even within the WHO 2021 IDH-wildtype GBM category; methylated tumors had longer median OS (15.7 vs 12.5 months), although in the adjusted Cox model MGMT methylation carried an adjusted HR 0.83 (95% CI 0.63-1.08), p = 0.162 and did not retain statistical significance after adjustment for age and Verhaak transcriptional subtype, consistent with the well-described confounding of MGMT status with age in unselected registry-style cohorts and with the principle that MGMT's most robust effect is seen in temozolomide-stratified analyses [13,14] rather than registry-style observational data. The further attenuation of the MGMT methylation effect after adjustment for any TMZ-containing therapy in our sensitivity analysis (HR 0.90, p = 0.457) is consistent with the established understanding that MGMT methylation's prognostic value in unselected GBM cohorts reflects, in part, its role as a predictor of TMZ benefit; in observational data where most patients receive TMZ, the marginal MGMT effect is diluted once treatment is accounted for, and the canonical MGMT effect is best demonstrated in treatment-stratified randomized trials.

Limitations

SEER [5] does not capture Karnofsky Performance Status, steroid use, extent of resection (beyond a surgery yes/no recode), TTFields use, MGMT status, or temozolomide dosing. Recorded chemotherapy in SEER is known to undercapture temozolomide use and should be interpreted as a proxy for systemic therapy rather than a temozolomide-specific indicator. Recorded receipt of surgery, radiation, and chemotherapy is subject to selection bias: healthier, younger, and better-performance-status patients are more likely to receive all three modalities. Patients with zero recorded survival months were excluded from time-to-event analyses; this is a standard analytic choice but may modestly bias estimates toward longer survival because SEER may code very-early deaths as zero months. The 145 SEER patients with unknown race (0.27% of the cohort) were excluded from the multivariable Cox model; this is a transparency rather than a substantive limitation given the small fraction affected. The TCGA cohort [7] is modest in size (n=319 in the multivariable model) and reflects academic contributing centers, which limits external generalizability. Methylation assays differed across TCGA contributing centers. The TCGA-GBM treatment field is heterogeneous, lacks dose and timing information, and includes "Unspecified Therapy" cases; our sensitivity analysis adjusting for any TMZ-containing therapy should therefore be interpreted as a coarse adjustment rather than a definitive treatment-stratified analysis, and TMZ receipt is itself confounded with patient fitness and other factors that determine treatment selection. We did not link individual SEER patients to TCGA tumors; the two cohorts are analyzed in parallel, not as a single integrated patient-level dataset. We did not adjust for recurrence patterns or second-line therapy, which increasingly affect post-progression survival in the TTFields era [6]. Proportional hazards assumptions were not formally tested.

## Conclusions

In glioblastoma, era-associated survival gains over the past two decades are only partially explained by uptake of recorded multimodality therapy, with a small residual era effect that persisted after adjustment for treatment receipt and measured clinical factors. MGMT promoter methylation was associated with improved unadjusted survival in IDH-wildtype TCGA-GBM but did not retain statistical significance after adjustment for age and Verhaak transcriptional subtype, consistent with the well-described confounding of MGMT status with age in unselected registry-style cohorts. Further population-level gains in GBM outcomes may require improved therapeutic strategies specifically for MGMT-unmethylated disease, which continues to account for the majority of patients and the bulk of deaths.
